# Very Low-Pressure CID Experiments: High Energy Transfer and Fragmentation Pattern at the Single Collision Regime

**DOI:** 10.3390/molecules29010211

**Published:** 2023-12-30

**Authors:** Dániel Szabó, Ágnes Gömöry, Krisztina Ludányi, Károly Vékey, László Drahos

**Affiliations:** 1 MS Proteomics Research Group, HUN-REN Research Centre for Natural Sciences, H-1117 Budapest, Hungarygomory.agnes@ttk.hu (Á.G.); vekey.karoly@ttk.hu (K.V.); 2 Department of Pharmaceutics, Semmelweis University, Hőgyes Endre 7–9, H-1092 Budapest, Hungary; ludanyi.krisztina@semmelweis.hu

**Keywords:** collision-induced dissociation (CID), energy transfer, low-pressure CID

## Abstract

We have performed CID experiments on a triple quadrupole instrument, lowering the collision gas pressure by 50 times compared to its conventional value. The results show that at very low-collision gas pressure, single collisions dominate the spectra. Indirectly, these results suggest that under conventional conditions, 20–50 collisions may be typical in CID experiments. The results show a marked difference between low- and high-pressure CID spectra, the latter being characterized in terms of ‘slow heating’ and predominance of consecutive reactions. The results indicate that under single collision conditions, the collisional energy transfer efficiency is very high: nearly 100% of the center of mass kinetic energy is converted to internal energy.

## 1. Introduction

Collision-induced decomposition (CID [1,2,3,4]) is one of the most frequently used fragmentation techniques in mass spectrometry. There are various experimental parameters influencing CID, like the collision energy (usually set in laboratory frame collision energy), type of the collision gas, pressure of the collision gas, the principle of ion storage, ion movement in the collision cell, geometry of the collision cell, residence time of ions inside the cell and even the energy content of ions entering the collision cell. In most studies, all of these parameters are set to a fixed value, usually to that suggested by the manufacturer. In fundamental studies, the collision energy is often varied [5,6,7]. Increasing the collision energy increases the internal energy of the colliding ions, and that in turn increases the degree of fragmentation, changes the ratio of various fragment ion abundances, and may also change fragmentation routes. Varying and optimizing the collision energy has various practical applications, such as determination of binding energies [8,9], differentiation of isomers [10,11,12], characterization of oligonucleotides [13] and small molecules [14,15,16,17], and various proteomic applications [18,19,20] including quantitation [21].

To date, effects of collision gas pressure on the CID spectra were investigated only in one case [22]. It was focused mainly on modeling, without addressing the possibility of single collisions. In this article, we aim to investigate the characteristics of single collision CID, with emphasis on its energetics, and fragmentation behavior. This is important to properly address fundamentals of CID and may open novel directions for instrument design that enables the utilization of unique fragmentation properties of presently unusual CID settings, such as the single collision pressure regimes presented in this work.

## 2. Results and Discussion

**Figure 1:**
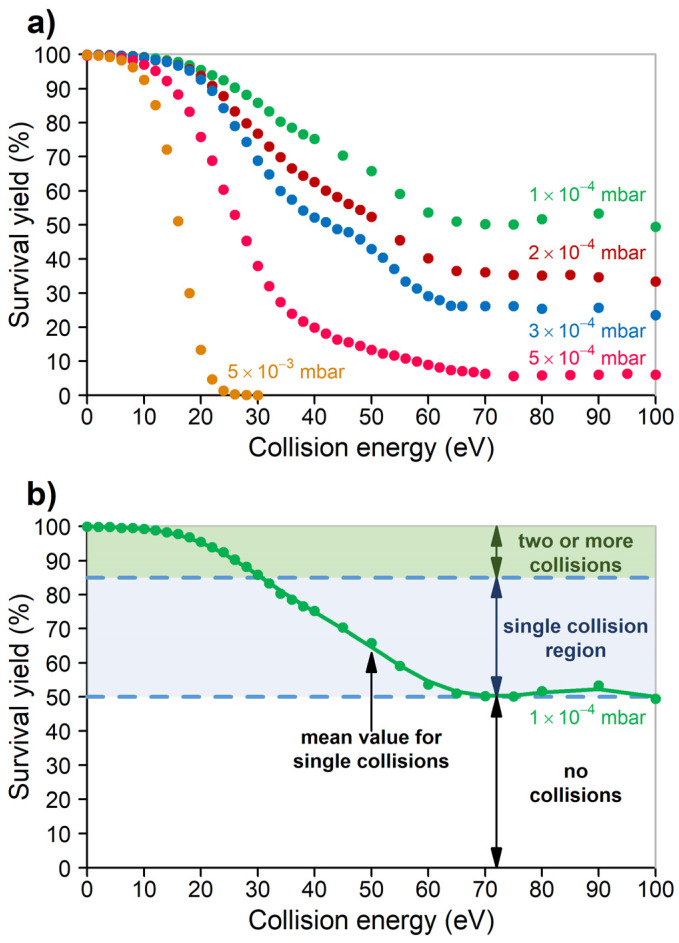
(**a**) Survival yield vs. collision energy curves at 5 different pressures from conventional (5 × 10^−3^ mbar) pressure to extremely low (1 × 10^−4^ mbar) for leucine enkephalin (using Ar collision gas). (**b**) Single collision region is in between 85 and 49% SY.

First, we have determined the survival yield (SY) curve [23,24] under conventional conditions. The survival yield is the fraction of intact parent ion compared to all ions:

SY = *I*_M_/(*I*_M_ + Σ *I*_F_) (1)

where *I*_M_ is the intensity (abundance) of the ionized molecule (in general, the precursor ion), while *I*_F_ is the fragment ion intensity (abundance). We use the SY, as it is a good indicator of the degree of excitation (measured by the internal energy) of the ions in the CID process [25,26,27]. Note, that if a fraction of the precursor ions does not collide, a modified version of this equation has to be used:

SY’ = (*I*_M_ − *I*_M_^∞^)/(*I*_M_ − *I*_M_^∞^ + Σ *I*_F_) (2)

where *I*_M_^∞^ represents the portion of molecular ions that do not fragment at all. Further considerations regarding this equation can be found in Appendix A. In this experiment, to model conventional conditions, we have used Ar collision gas and 5 × 10^−3^ mbar pressure indicated on the ion gauge, as suggested by the manufacturer. The result is shown in Figure 1a (yellow points), analogous curves have often been published [7,13,22,25,28]. This shows that increasing the (laboratory frame) collision energy to ca. 30 eV provides sufficient internal energy to the MH^+^ ions, so that all of them fragment before leaving the collision cell. Note that we use a commercial triple quadrupole instrument; the ion gauge is close to the vacuum pump, so it does not measure the pressure directly in the collision cell. The actual pressure in the collision cell can be estimated, based on the average number of collisions (estimated based on the ratio of non-colliding parent ions), the length of the collision cell, and the collisional cross section of leucine enkephalin. 

Decreasing the collision gas pressure by a factor of 10 (which is far lower than that typically used; Figure 1a, red curve), the SY curve changes: most significantly, to reach the same degree of fragmentation as before, ca. 70% higher collision energy is required. The explanation is straightforward: at lower pressure, there are fewer collisions, so these need to be of higher energy to reach the same degree of excitation. There is a further difference: above ca. 70 eV, there is a small fraction of parent ions (ca. 6%), which do not fragment however high we increase the collision energy. This becomes more pronounced when we further decrease the collision gas pressure to 1 × 10^−4^ mbar (Figure 1, green curve): now, fragmentation requires still more collision energy, and ca. 49% of the parent ions do not fragment even at a very high collision energy. This is explained by assuming that at 1 × 10^−4^ mbar pressure 49% of the incoming ions pass through the cell without colliding at all. Note, that 5 × 10^−4^ mbar vacuum gauge readout corresponds to a ratio of 6% of non-colliding precursor ions, and the real pressure in the collision cell is estimated to be 1.4 × 10^−3^ mbar. At 1 × 10^−4^ mbar readout, the proportion of the non-colliding precursor ions is 49%, and the real pressure in the cell is estimated to be 3.5 × 10^−4^ mbar.

**Figure 2:**
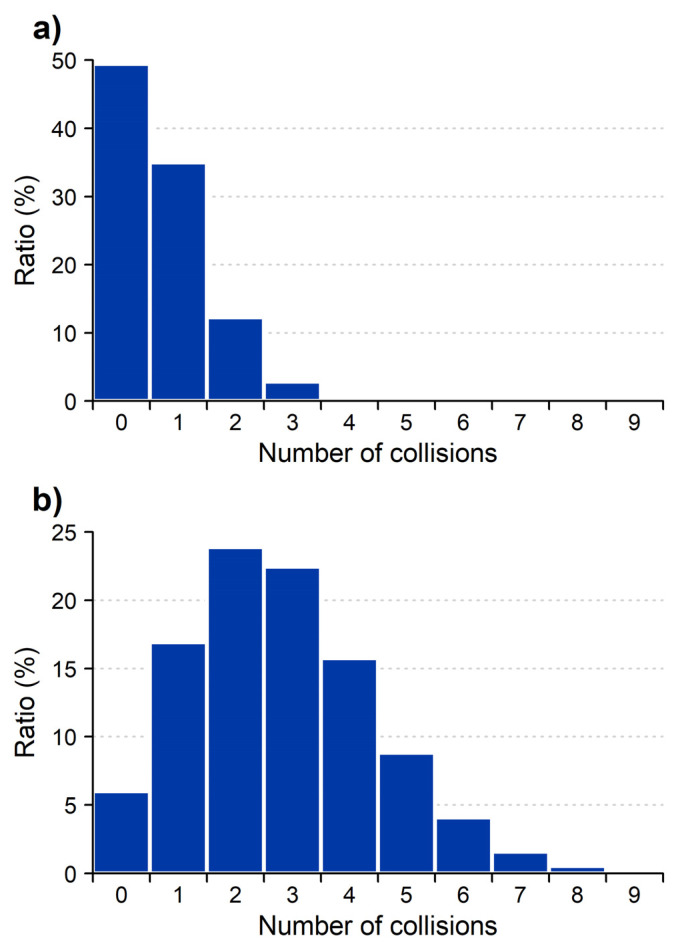
Distribution of multiple collision probabilities at (**a**) 1 × 10^−4^ and (**b**) 5 × 10^−4^ mbar pressure (vacuum gauge readout).

These experiments may be used to determine the average number of collisions both at low pressure and under conventional conditions. Determining the average number of collisions requires only combinatorial calculation, although we have performed the calculations using the MassKinetics 2.1 [29] software. At 1 × 10^−4^ mbar pressure (Figure 1, green curve), at high energy, 49% of the incoming ions do not collide. The average number of collisions is 0.70, single collisions have 35%, double collisions 12%, triple collisions 3% and quadruple collision 0.5% probability. This distribution is shown in Figure 2.

At 5 × 10^−4^ pressure (Figure 1a, red curve) and at high energy, 6% of the incoming ions do not collide. The average number of collisions is 2.81, the probability distribution is shown in Figure 2b. The manufacturer suggests collision gas pressure readings in the operative range of the instrument to be 3–8 × 10^−3^ mbar. As the collision gas pressure and the number of collisions correlate linearly, this suggests that, on average, ca. 20–50 collisions occur in a typical quadrupole CID experiment for leucine enkephalin. It is significantly higher than that suggested in the literature, even if the collision cross section of various ions is also taken into account.

**Figure 3:**
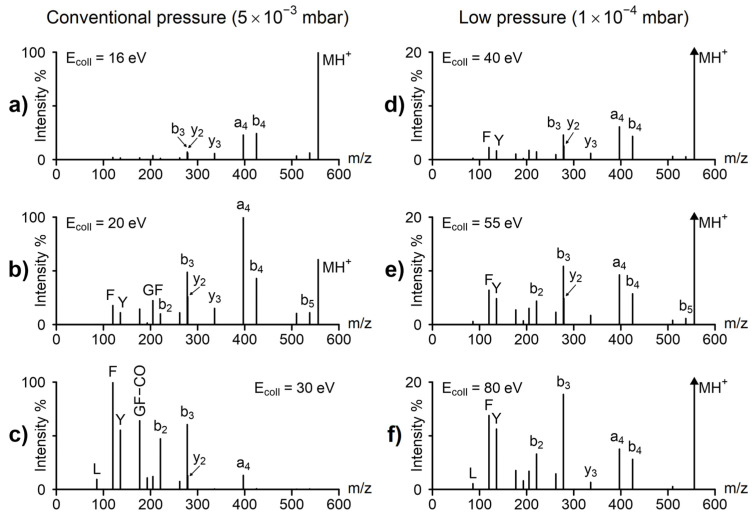
Tandem mass spectra of leucine enkephalin at conventional pressure (5 × 10^−3^ mbar vacuum gauge readout) at (**a**) 16 eV, (**b**) 20 eV, (**c**) 30 eV collision energy; and at low pressure (1 × 10^−4^ mbar vacuum gauge readout) at (**d**) 40 eV, (**e**) 55 eV, (**f**) 80 eV collision energy. Collisional energies at conventional pressure were selected to demonstrate various degrees of fragmentation, while the low-pressure collision energies were selected so that the spectra would be most similar to those of the corresponding conventional pressure.

Up to now we have discussed the effect of collision gas pressure on the survival yield, but low pressure changes the spectra as well—both the fragmentation processes and the fragment ion ratios are changed. Figure 3 shows the energy-dependent spectra of leucine enkephalin at conventional and at a very low pressure, 5 × 10^−3^ and 1 × 10^−4^ mbar, respectively. For the comparison in Figure 3, collisional energies at conventional pressure were selected to demonstrate various degrees of fragmentation, while the low-pressure collision energies were selected so that the spectra would be most similar to those of the corresponding conventional pressure. Note, that at low pressure, spectra at the 16–30 eV range show practically no fragmentation, while conventional pressure spectra at 40–80 eV show only low mass fragments. The low energy, conventional pressure spectrum (Figure 3a) is taken at 16 eV collision energy, corresponding to 49% survival yield. This spectrum is dominated by low energy fragments (like a_4_, b_4_). At this pressure, there are many low-energy collisions, and these result in predominantly low-energy fragments (‘slow heating’ [30], like that observed typically in ion traps). This spectrum can be compared to the low-energy, low-pressure spectrum, taken at 40 eV, shown in Figure 3d. Note that at low pressure (1 × 10^−4^ mbar), single collisions dominate (Figure 2a), and 16 eV collision energy is insufficient to cause fragmentation; 40 eV collision energy was selected, as this is the collision energy, where 51% of the colliding ions fragment. The low-energy, low-pressure spectrum (Figure 3d) contains both low-energy (a_4_, b_4_) and high-energy (b_3_, F, Y) fragments and is therefore significantly different from the low-energy, high-pressure spectrum (Figure 3a). At low pressure, there is only a single, higher energy collision, and this gives rise to both low- and high-energy fragments. Fragmentation in the single collision regime means that the incoming ion is excited in one step, i.e., the internal energy goes up to relatively high values above the fragmentation threshold. It is in contrast with the slow heating regime [30], where the multistep excitation results in the fragmentation of ions just above the fragmentation threshold. This difference in mechanism makes it possible for high-energy processes to appear in the spectra under single-collision conditions.

Significantly increasing the collision energy ca. 1.5 and 2 times at low pressure (Figure 3d,e) changes fragmentation only moderately: the abundance of the protonated molecule is decreased, but due to the large fraction of non-colliding ions, it is the most abundant ion even at the highest energy (Figure 3f). The same fragment ions are present both at low and at high energy; only the fragment ion ratios change by a moderate degree. This change may be characterized by the abundance ratio of the low-energy b_4_ + a_4_ ions compared to that of the high-energy Y and F ions. At 40 eV (Figure 3d), it is ca. 3:1, while at 80 eV, it is about 1:2. These differences are consistent both with increasing the internal energy with increasing the collision energy, and with the expected wide internal energy distributions caused by the predominantly single collision regime. 

At conventional pressure (Figure 3a–c), the influence of the collision energy is much larger. Both the ionized molecule and the first-stage, low-energy fragments (a_4_, b_4_) quickly disappear with increasing collision energy, and the spectra are dominated by low-mass fragments formed in a consecutive reaction sequence (like b_4_ >> a_4_ >> b_3_ >> Y). The abundance ratio of the low energy a_4_ + b_4_ ions to that of the high-energy F + Y ions is particularly revealing: at low energy (16 eV, Figure 3a), it is 14:1, while at 30 eV (Figure 3c), it is 1:11, the difference being about 150-fold. This large difference can be explained, again, by the ‘slow heating’ mechanism. Note, there is a major difference to ion trap fragmentation: in quadrupoles, not only the precursor ion but all fragment ions participate in heating collisions. This means that the MH^+^ ion is ‘slow heated’ and forms, e.g., the b_4_ ion. These (fast-moving) b_4_ ions collide further, are re-excited, and are fragmenting in a second, low-energy, process to yield the b_3_ ion. And this procedure may be repeated several times. This process, in contrast to the low-pressure, single-collision regime, favors consecutive low-energy fragmentation steps (fragmentation cascades). 

For analytical purposes, “conventional pressure” CID is often advantageous, as the spectra show a large variety of fragments. The low-pressure CID, on the other hand, is much better for mechanistic studies, as most ions are formed in single-stage processes and therefore carry more important structural information. Furthermore, low-pressure, high-energy spectra may contain special high-energy fragments, like charge remote fragmentation observed in sector instruments [31,32,33]. Such fragmentation processes often provide direct information on the structure of the studied compound unavailable under low-energy (slow-heating) conditions.

Low-pressure CID at single-collision conditions may also be used to derive information on kinetic to internal energy conversion (energy transfer efficiency). The maximum amount of kinetic energy, which may be converted into internal energy in a single collision, is the center-of-mass collision energy (*E*_com_). The fraction of *E*_com_ converted into internal energy is typically called the (kinetic to internal) energy transfer efficiency. Estimates relating to the quadrupole collision energy regime range from 10 to 100% [34,35,36,37]. This wide range implies high uncertainty in the estimation collision energy transfer efficiency. There are relatively few reliable experiments, performed under various conditions, often on specially built instruments [36], which makes reproduction and verification problematic. Evaluation of these experiments usually requires advanced theoretical calculations. Such advanced studies are typically performed by research groups working in fundamental studies and are highly informative regarding not only the average energy transfer but also its distribution. However, these studies are very difficult to reproduce independently, and the inherently complex data evaluation scheme may contain unknown error sources. Furthermore, such experiments are difficult to compare directly and, consequently, their results often seem to be contradictory.

The low-pressure quadrupole experiments discussed above provide a means to estimate collisional energy transfer. In principle, there are two possible options. One could carry out accurate calculations or provide simple estimates. Both have advantages and disadvantages. Accurate modelling of SY curves should provide a good estimate of the energy transfer efficiency. However, such calculations are very complicated, and would require specialized software (like Masskinetics 2.1 [29] or CRUNCH [36]). The disadvantage of using such complex models is that the calculations are nearly impossible to control independently. Even with the best intentions, they may contain unexpected errors or may use wrong assumptions. 

The alternative approach is to use simple models, which are easy to examine and control independently. We aimed to keep the experiments and calculations as simple as possible. This has several advantages: (1) experiments are simple to reproduce as they require commercially available instrumentation only and are easy to extend to other compounds or collision gases. (2) The concept of the calculations presented is unambiguous and can be verified by other researchers. They also require only a few assumptions and few parameters, as described below. (3) Due to the simple experimental design and straightforward evaluation, possible errors are easy to identify and to avoid. (4) The results will be reliable, even if not accurate. The disadvantages are that the results will give information on the average energy transfer efficiency (and not its distribution), and the results will not be very accurate. 

Among the experimental results discussed above, here, we use the results at 1 × 10^−4^ mbar pressure (Figure 1b). Note that 49% of the ions do not collide (and therefore do not fragment even at high energy). A total of 15.5% of the ions collide more than once (as discussed above), and, therefore, these fragment at the lowest collision energies (see Figure 1b, green region, SY = 84.5–100%). The rest, between 84.5% and 49% survival yield, corresponds to single collisions (Figure 1b, blue region). The mid-point between these two values (at 67% SY) corresponds to the case, when 50% of the singly colliding ions fragment (Figure 1b). The collision energy corresponding to this survival yield is 50 eV (Figure 1). Using the simple equation

*E*_com_ = *E*_lab_ × *m*_gas_/(*m* + *m*_gas_) (3)

the center-of-mass (com) collision energy can be determined ($m_{gas}$ is the mass of the collision gas, m is the mass of the precursor ion and $E_{lab}$ is the laboratory frame kinetic energy) at the 50 eV laboratory frame collision energy corresponding to *E*_com_ = 3.39 eV. Note the slight shoulders in the SY curves in Figure 1 between 40 and 60 eV, especially visible at 3 × 10^−4^ pressure. These correspond to fragmentations due to a single collision. Due to the wide internal energy distributions (typical in most mass spectrometric experiments), the influence of single and multiple collisions on the SY curves is not well resolved. 

When no collision gas is used, it is possible to determine the degree of spontaneous fragmentation (these would be the “metastable” ions in a sector instrument). It was found to be 0.03%, measuring the SY curve with no collision gas (only background gas) and extrapolating these to zero-collision voltage. 

The rate of fragmentation of protonated leucine enkephalin, as a function of internal energy, was calculated using straightforward Rice–Ramsperger–Kassel–Marcus (RRKM) calculations. Parameters for the RRKM calculations of leucine enkephalin fragmentation (critical energy (*E*_0_ = 1.19 eV), molecular and transition state frequencies) have been taken from the literature [38]. The residence time in the collision cell is determined from the collision voltage (determining the speed of ions) and the length of the collision cell (5 cm). At 50 V collision voltage, it is 12 μs. 

We evaluate the data using two sets of approximations. The simplest is to use average energies and ignore distributions. First, we determine the amount of internal energy corresponding to the observed spontaneous fragmentation, and this is the average internal energy of the protonated molecules entering the collision cell. Second, we determine the amount of internal energy needed to result in a 51% fragmentation rate inside the collision cell. The difference between these two values is the amount of internal energy gained in the collision cell. The ratio between the energy gained in the collision and *E*_com_, the maximum amount of energy that can be converted into internal energy in a single collision, is the energy transfer efficiency. 

Using straightforward RRKM [39,40,41] calculations, in the case of leucine enkephalin, 4.20 eV internal energy will cause 0.03% spontaneous fragmentation in the collision cell—and this is the internal energy of ions entering the collision cell. The internal energy causing 51% fragmentation is 8.45 eV. These numbers indicate that 4.25 eV kinetic energy needs to be converted to internal energy to explain 51% fragmentation. On the experimental side, for single collisions (Figure 1b), 50 eV laboratory frame collision energy (3.39 eV *E*_com_) is needed to fragment 51% of the ions. Comparing the *E*_com_ (3.39 eV) to the 4.25 eV needed to cause this degree of fragmentation indicates 125% collisional energy transfer. Note, 100% is the theoretical maximum, so this approximation overestimates the energy conversion efficiency. We know it is a very large overestimation, but it reflects that it is a simple model. Accuracy in the ± 50% range might be expected. What we find very important is that the energy transfer efficiency is very high, close to 100% and far higher than 10%, as suggested before. 

The second set of approximations assumes thermal energy distributions, both for ions entering the collision cell and for ions after the collision. In the mass spectrometer, ions often have close to thermal energy distributions [42,43], so it is a more reasonable assumption than using averages only. However, this is still an approximation only. RRKM calculations using thermal energy distributions indicate that ions at 602 K temperature will result in 0.03% spontaneous fragmentation. The mean internal energy at 602 K is 3.68 eV for leucine enkephalin (note, this is close to that of 4.20 eV estimated based on averages only); 51% fragmentation is observed at 967 K, corresponding to 7.82 eV mean thermal energy. Using this approximation, the energy increase in the collision is 4.14 eV. This corresponds to 122% kinetic to internal energy conversion, which is also an overestimate. There are two important consequences of this calculation. The first is that both simple models indicate high, close to 100%, translational to internal energy conversion in a single collision. The second conclusion is that energy distributions have relatively little effect on the calculation, changing the end result by only 3%. Note, that effective temperatures in the range of 500–1000 K are considered typical in fragmentation studies [44,45].

In the discussion above, we think that there are three main error sources. Below, we summarize these and estimate their influence on the energy conversion efficiency. First, there are errors with respect to the initial internal energy distribution. We can estimate its influence by varying the degree of spontaneous fragmentation. Second, the residence time in the collision cell may be erroneously estimated and may depend on various tuning parameters. Furthermore, the residence time of the ions in the collision cell depends on the kinetic energy of the ions, and this effect has been neglected above. Third, the RRKM calculation of the reaction rate may also be in error. We may estimate its importance by varying the critical (or activation) energy. The effects of these parameters are summarized in Table A1 found in Appendix B, calculated using thermal energy distributions, as discussed above. As shown in Table A1, estimates for energy conversion efficiency all indicate very high values, ranging from 91% to 142% (note, the theoretical limit is 100% maximum). There is a further major error source: the shape of the internal energy distribution. Its influence is difficult to numerically estimate. The result, that the difference between the estimates based on thermal distribution and no distribution at all is relatively small, suggests that its influence is not critical. 

**Figure 4:**
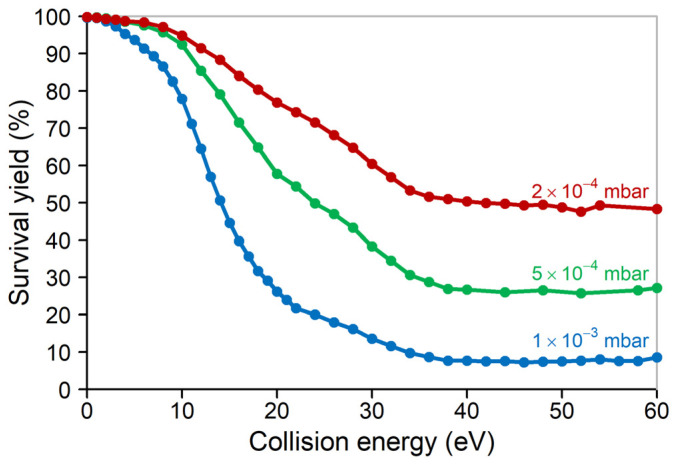
Survival yield curve of the sodium formate cluster (HCOO)_5_Na_6_^+^ using Ne collision gas at 3 different pressures (pressure gauge readout).

We have also studied other compounds and other collision gases as well, and they have shown analogous results using very low-pressure CID. In Figure 4, we show collision gas pressure-dependent survival yield curves for the sodium formate cluster (HCOO)_5_Na_6_^+^ using Ne collision gas. Qualitatively, it is very similar to those of leucine enkephalin (Figure 1). Sodium formate clusters have been studied before; their structure, energetics and frequency files corresponding to their fragmentation are available [46]. Using these data, we have performed a similar RRKM modelling for the (HCOO)_5_Na_6_^+^ cluster as that discussed above for leucine enkephalin. The sodium formate cluster showed very small, ca. 0.014%, spontaneous fragmentation. Assuming thermal energy distributions, this corresponds to 526 K temperature and 1.62 eV mean internal energy. The temperature corresponding to 51% fragmentation probability is 826 K (the mean internal energy is 2.62 eV). At 2 × 10^−4^ mbar pressure, ca. 49% of the precursor ions do not fragment even at high collision voltage. As discussed above, at this pressure 67% survival yield is the point, where 50% of the singly colliding ions fragment. This survival yield corresponds to 26 eV laboratory frame (and 1.36 eV com frame) collision energy (Figure 4). The internal energy increase according to this model is 2.61–1.62 = 1.00 eV; suggesting 1.00/1.36 = 74% kinetic to internal energy conversion. Although this value is lower than that observed for leucine enkephalin, it is nevertheless very high efficiency. The difference might be due to the smaller (less polarizable) collision gas (Ne vs. Ar), or to the different ion structure, or to errors in one or both models.

## 3. Materials and Methods

Leucine enkephalin (amino acid sequence is YGGFL) and MS-grade formic acid were purchased from Sigma-Aldrich (Budapest, Hungary). HPLC-MS grade water and acetonitrile were purchased from VWR International Kft.

Experiments were performed using a Waters Micromass Quattro (Manchester, UK) mass spectrometer in positive electrospray ionization mode. The samples were infused with a syringe pump into the electrospray source at the rate of 20 μL/min using 1:1 water:acetonitrile + 0.1% formic acid as solvent. With the exception of the collision gas pressure and collision energy, experimental conditions were kept constant during the experiments. The source conditions were as follows: voltage of the capillary was 2.7 kV, the voltage of the cone was 40 V, and the temperature of the source was 373 K. The collision gas was argon (5.0 purity, purchased from Linde Magyarország Kft). The collision energy was varied in the 0–100 eV range, and the pressure of collision gas was varied in the 1 × 10^−4^–5 × 10^−3^ mbar range (as indicated by the vacuum gauge).

## 4. Conclusions

Decreasing the operational pressure in the CID cell by 50–100 times results in a pressure range, where single collisions dominate. The spectra under such conditions contain both low- and high-energy fragments. Single-step fragmentation processes dominate the spectra, even at high energy. The low-pressure CID spectra show some analogies with keV collisions on sector instruments as well. Both cases operate in the single-collision regime, and in both cases, there are ions with high internal energy (in contrast to slow heating methods). The results highlight that CID under conventional conditions is the result of a high number of collisions, even as many as 20–50 collision events. In proteomics, it has become fashionable to use ‘composite spectra’, which are a combination of low- and high-energy CID spectra [18,20,21,47]. The result is qualitatively similar to the low-pressure CID spectra studied here.

The extremely low-pressure CID spectra may be especially useful for fundamental studies. Here, we demonstrated that the results give an indication of the collisional (kinetic to internal) energy transfer. The results show very high, close to 100%, energy transfer efficiency in single collisions. The calculations were intentionally kept very simple: on the one hand, these are easy to control and reproduce—which attests that the very high collisional energy transfer efficiency is a reliable estimate. On the other hand, simple calculations do not provide high accuracy. We are in the process of a detailed reaction kinetics modelling to address the problem of accuracy.

## Data Availability

Data are contained within the article.

## Appendix A

### Consideration of Non-Colliding Molecular Ions

When a fraction of parent ions does not collide due to the low pressure (estimated by the fraction of molecular ions, which do not fragment even at very high energy), it is worth introducing a definition of modified survival yield that does consider the molecular ions that do not collide:

SY’ = (*I*_M_ − *I*_M_^∞^)/(*I*_M_ − *I*_M_^∞^ + Σ *I*_F_) (A1)

where *I*_M_^∞^ represents the portion of molecular ions that do not fragment at all, which theoretically should be equal to the intensity of molecular ions at infinitely high collision energy. Note, at conventional pressure the SY and SY’ curves are identical.

## Appendix B

**Table A1:** Changed parameters to estimate the error of energy transfer.

Parameter	Values	Energy Conversion (eV)	Energy Transfer
Degree of spontaneous fragmentation	0.03%	3.74	131%
	0.10%	4.44	110%
Residence time for spontaneous fragmentation	6 μs	3.91	115%
	24 μs	4.34	128%
Residence time for CID fragmentation	6 μs	4.82	142%
	24 μs	3.08	91%
Critical energy	1.09 eV	3.62	107%
	1.29 eV	4.77	141%
